# Impact of elevated systolic arterial pulmonary pressure on the total mortality rate after acute myocardial infarction in the elderly

**DOI:** 10.1038/s41598-022-16210-6

**Published:** 2022-07-23

**Authors:** Salim Bary Barywani, Magnus C. Johansson, Silvana Kontogeorgos, Zacharias Mandalenakis, Per-Olof Hansson

**Affiliations:** 1 Department of Molecular and Clinical Medicine, Institute of Medicine, Sahlgrenska Academy, University of Gothenburg, Gothenburg, Sweden; 2 Department of Medicine, Geriatrics and Emergency Medicine, Region Västra Götaland, Sahlgrenska University Hospital, Gothenburg, Sweden; 3 Department of Clinical Physiology, Region Västra Götaland, Sahlgrenska University Hospital, Gothenburg, Sweden

**Keywords:** Medical research, Outcomes research

## Abstract

Reduced left ventricular ejection fraction (LVEF) is associated with increased mortality after acute myocardial infarction (AMI). However, the prognostic impact of elevated systolic pulmonary artery pressure (sPAP) in the very elderly patients after AMI is lacking. We aimed to study the impact of elevated sPAP on one- and five-year all-cause mortality after AMI in very elderly patients, 80 years of age and older. Of a total number of 353 patients (≥ 80 years) who were hospitalized with acute coronary syndrome, 162 patients presenting with AMI and with available data of sPAP on echocardiography were included and followed-up for 5 years. The survival analyses were performed using Cox-Regression models adjusted for conventional risk factors including LVEF. Altogether 66 of 162 patients (41%) had ST-segment elevation MI, and 121 (75%) of patients were treated with percutaneous coronary intervention in the acute phase. Echocardiography during the admission revealed that 78 patients (48%) had a LVEF ≤ 45% and 66 patients (41%) had a sPAP ≥ 40 mmHg. After one and five years of follow-up, 23% (n = 33) and 53% (n = 86) of patients died, respectively. A multivariable Cox-Regression analysis showed that the elevated sPAP (≥ 40 mmHg) was an independent predictor of increased mortality in both one and five years after AMI; HR of 2.63 (95%, CI 1.19–5.84, *P* 0.017) and HR of 2.08 (95%, CI 1.25–3.44, *P* 0.005) respectively, whereas LVEF ≤ 45% did not show any statistically significant impact, neither on one- nor on five-year mortality (HR 1.3, 95% CI 0.6–2.9, *p* = 0.469) and (HR 1.4, 95% CI 0.8–2.4, *p* = 0.158), respectively. Elevated sPAP was an independent risk factor for one- and five-year all-cause mortality after AMI in very elderly patients and sPAP seems to be a better prognostic predictor for all-cause mortality than LVEF. The risk of all-cause mortality after AMI increased with increasing sPAP.

## Introduction

Heart failure (HF) after acute myocardial infarction (AMI) is associated with increased mortality and morbidity in all age groups^1–9^. Previous studies have identified several risk factors for impaired survival after AMI, including reduced left ventricular ejection fraction (LVEF) as a marker for structural left ventricular systolic dysfunction^10–13^. Several studies have demonstrated also the impact of elevated sPAP after AMI as an independent risk factor of increased all-cause mortality. These studies have included relatively young patients with relatively short-term follow-up. For instance, Delgado and co-authors^14^in their well-designed study included patients with an average age of 65 years with a follow-up period of 1 year, Møller and co-authors^15^ in their study included patients with an average age of about 74 years with a follow-up period of 40 months and Fan and co-authors^16^ in their study included patients with an average age about 65 years and a follow-up period of only 6 months. Mutlak and co-authors^17^ performed a prospective longitudinal observational study designed to determine predictors of post myocardial infarction HF, and they demonstrated that 44% of the patients had a systolic pulmonary artery pressure (sPAP) > 35 mmHg. In addition, elevated sPAP at the index admission was a useful marker in unmasking latent subclinical HF and predicting the development of overt HF. Thus, the available evidence on the impact of sPAP on survival after AMI is mainly conducted in relatively young patients (< 80 years) with a short terms follow-up. To the best of our knowledge, the prognostic impact of elevated sPAP on mortality in very elderly patients is not studied. In the present study we sought to investigate the impact of elevated sPAP on short-and long-term all-cause death after AMI in elderly patients ≥ 80 years.

## Methods

### Study cohort

Three hundred and fifty-three patients aged ≥ 80 years hospitalized due to acute coronary syndrome at the two major Cardiology departments at Sahlgrenska University Hospital (SU), (SU/Sahlgrenska and SU/Östra), affiliated with the University of Gothenburg during 2006–2007 were included consecutively. All patients presented with acute myocardial infarction and examined with echocardiography with adequate data on sPAP were included in the study and retrospectively followed up regarding all-cause death for5 years (*n* = 162). Treatment with percutaneous coronary intervention (PCI) or not was based on a pure clinical decision made by the attending cardiologist. All PCIs were performed at a joint PCI center for both hospitals. Patients were retrospectively identified from the hospital patient registry. Patients from other hospitals who were referred to SU only for PCI were excluded due to incomplete follow-up data. The study protocol was approved by the Ethical Committee at the University of Gothenburg.

### Echocardiography

All echocardiography examinations were performed at the department of Clinical Physiology at Sahlgrenska University Hospital by trained echo technicians or physicians according to standardized protocol. Echocardiography examinations were performed after coronary angiography examinations in patients presented with STEMI and before coronary angiography examinations in patients presented with non-STEMI. However, all echocardiography studies were performed before discharge from the hospital. Echocardiography data including data on sPAP were retrieved from the echocardiography reports already performed for clinical purposes. LVEF was measured using Simpson biplane or when not feasible by visual estimate. Left atrial area was measured in apical 4 chamber view. LV filling pressure was qualitatively estimated by pulsed wave Doppler of mitral and pulmonary vein flow as increased or normal^18^. Mitral regurgitation was analyzed by colour and continuous wave Doppler and considered present when there more than trace regurgitation. Aortic stenosis was considered present when the peak gradient was ≥ 25 mmHg. Reference values were those used in clinical praxis and were based on results from a previously examined local healthy population sample from which regression equations had been constructed based on sex, body surface area, weight and age. The equations were incorporated in a spreadsheet, resulting in a z-score and an increased parameter-value for an individual was defined as a z-score ≥ 2.0. The reference limits where thus individually calculated for each patient.

### Estimation of systolic pulmonary artery pressure

Systolic pulmonary artery pressure (sPAP) was estimated by echocardiography from the tricuspid valve regurgitant jet velocity using the modified Bernoulli equation 4v^2^ plus right atrial pressure^19,20^. Special care was taken to align the Doppler cursor with the tricuspid regurgitation jet. Right atrial (RA) pressure was estimated from characteristics of the inferior vena cava (IVC); based on the diameter and the respiratory variation in the diameter of the IVC: an IVC diameter ≤ 21 mm that collapses > 50% with a sniff suggested a normal RA pressure of 5 mmHg, whereas an IVC diameter ≤ 21 mm that collapses < 50% or an IVC diameter > 21 mm that collapses > 50% with a sniff regarded as an intermediate value, 10 mmHg. AIVC diameter > 21 mm that collapses < 50% with a sniff or < 20% on quiet inspiration regarded as a high RA pressure of 15 mmHg.

### Statistics

Categorical variable were described as percentages and compared using chi-square test or Fisher exact as appropriated. Continuous variables were described as means ± standard deviation (SD) and compared using independent sample test or One-way analysis of covariance. To adjust for the underlying baseline characteristics and to analyze for probable association between different levels of the sPAP and mortality, the cohort was analyzed using multivariable Cox proportional-hazard regression models analyzing time to event. The results were adjusted for all the baseline variables demonstrated in Tables 1 and 2 systematically, according to the principles of Cox-regression model building. That means the variables were first included in univariable models and only variables with a *p*-value < 0, 05 have been included in the multivariable models which included 8 variables indicating about 11 events (86 events) per variable. To identify the lowest cut off level of the sPAP associated with increased mortality rate, multivariable models were built for different sPAP levels, as low as 30 mmHg and upward with 5 mmHg intervals. The multivariable Cox models were assessed for proportional hazard assumption for covariates graphically with adjusted log minus log curves. The hazard ratios (HRs)with confidence intervals (CIs) and *p*-values were presented. All statistical analyses were performed using SPSS 22 statistical software. *P*-value < 0.05 was regarded as statistically significant.

**Table 1:** Demographic and clinical characteristics of the study patients, comparing patients with systolic arterial pulmonary pressure (sPAP) ≧40 mmHg and sPAP < 40 mmHg.

	sPAP ≧ 40 mmHg (n = 66)	sPAP < 40 mmHg (n = 96)	*P*-value
**Demographics**			
Age, year	84.4 ± 2.8	83.7 ± 2.8	0.089
Gender, male	32 (48.5)	58 (60.4)	0.133
Weight, kg	70.7 ± 15.4	73.2 ± 12.0	0.265
Height, cm	167.6 ± 22.2	169.5 ± 8.8	0.452
BMI, kg/m^2^	23.6 ± 3.7	25.4 ± 3.8	0.004
Smoking, yes	6 (9.5)	5 (5.3)	0.321
**Clinical characteristics**			
STEMI, yes	35 (53)	38 (39.6)	0.747
Non-STEMI, yes	31 (47.0)	58 (60.0)	0.091
PCI, yes	44 (66.7)	77 (80.2)	0.051
Heart rate, bpm	81.8 ± 16.7	80.8 ± 29.7	0.881
Systolic BP, mmHg	145.6 ± 28.6	150.4 ± 26.6	0.296
Diastolic BP, mmHg	85.8 ± 16.1	82.8 ± 16.1	0.271
**Laboratory findings**			
Hemoglobin, g/L	129.8 ± 15.2	131.7 ± 17.0	0.478
eGFR, ml/min/1.73m^2^	49.1 ± 19.7	50.3 ± 18.3	0.682
Creatinine, umol/L	110.0 ± 104.2	110.7 ± 77.6	0.961
**Comobidities**			
Atrial fibrillation, yes	17 (27.0)	17 (17.7)	0.153
History of heart failure, yes	14 (23.0)	17 (17.7)	0.355
Hypertension, yes	35 (53.0)	40 (41.7)	0.103
Diabetes, yes	13 (21.0)	16 (17.6)	0.374
Hyperlipidaemia, yes	9 (13.8)	8 (8.3)	0.195
Previous stroke, yes	11 (19.6)	10 (11.0)	0.284
**Medications**			
β- Blockers, yes	34 (51.5)	51 (56.7)	0.317
ACEI/ARB, yes	20 (30.3)	26 (27.1)	0.517
diuretics, yes	16 (24.2)	19 (20.9)	0.378
Calcium channel blocker, yes	14 (21.2)	36 (39.1)	0.017
Statins, yes	11 (16.7)	21 (22.6)	0.238
Digoxin, yes	9 (15.0)	10 (10.8)	0.296

*BMI* Body mass index, *STEMI ST*-elevation myocardial infarction, *Non-STEMI* Non-ST-elevation myocardial infarction, *PCI* Percutaneous coronary intervention, *BP* Blood pressure, *eGFR* Estimated glomerular filtration rate, *ACEI* Angiotensin converting enzyme inhibitors, *ARB* Angiotensin receptor blockers.

**Table 2:** Echocardiographic characteristics of study patients, comparing patients with systolic arterial pulmonary pressure (sPAP) ≥ 40 mmHg and sPAP < 40 mmHg.

	sPAP≧40 mmHg (n = 66)	sPAP < 40 mmHg (n = 96)	*P*-value
Left ventricular ejection fraction, %	41.7 ± 10.6	49.5 ± 10.4	< 0.001
Elevated left ventricular filling pressure, yes	29 (43.9)	16 (21.6)	< 0.001
Dilated left ventricle, yes	16 (25.4)	15 (16.5)	0.125
Dilated left atrium, yes	32 (54.2)	29 (30.2)	0.008
Mitral valve regurgitation ≥ grad 1/4, yes	37 (56.9)	23 (25.0)	< 0.001
Tricuspid valve regurgitation ≥ grad 1/4, yes	9 (13.8)	4 (4.4)	0.036
Aortic valve stenosis, yes	16 (27.6)	11 (13.3)	0.029

*sPAP* Systolic pulmonary artery pressure.

### Laboratory analysis

All laboratory variables were analyzed, according to routine protocol, by the Clinical Chemistry Laboratory at Sahlgrenska University Hospital. Cockcroft–Gault formula was used to estimate the glomerular filtration rate (eGFR) in ml/min/1.73m^2^.

### Clinical outcome data

The primary endpoints were one-and five-year all-cause mortality after acute myocardial infarction. Data on time of death were obtained from the Swedish Cause of Death registry which includes all deaths of persons registered in Sweden.

### Ethics approval and consent to participate

The Ethical Committee at the University of Gothenburg approved and granted permission to access and use the medical records described in the study. The ethical committee also ruled that no formal consent was necessary. The protocol was performed in accordance with the relevant guidelines and regulations according to the declaration of Helsinki.

## Results

### Clinical characteristics

Of the whole cohort, about 41% of the patients had STEMI and 59% had non-STEMI with an average left ventricular ejection fraction (LVEF) about 46%. The target vessel was left descending coronary artery (LAD) in about 49% of the patients, circumflex coronary artery (Cx) in 14% and right coronary artery (RCA) in 11%. About 40% of the patients had anterior myocardial infarction (MI), 14% had inferior MI and 46% had MI with unspecified localization. The clinical characteristics of the patients, comparing patients with sPAP ≥ 40 mmHg with the patients with sPAP < 40 mmHg are presented in Table 1. The sPAP ≥ 40 mmHg was the lowest sPAP level associated with increased mortality. Patients with sPAP  ≥ 40mmHg had lower BMI (23.6 ± 3.7 vs. 25.4 ± 3.8 kg/m^2^) and were less frequently treated with calcium channel blocker (21.2% vs. 39.1%) compared to patients with a sPAP < 40mmHg.There were no significant differences between the two groups in gender, previous stroke or in number of patients treated with PCI, betablockers, angiotensin converting enzyme inhibitors/angiotensin receptor blockers, diuretics, statins, or digoxin.

### Echocardiography findings

Compared to patients with sPAP < 40 mmHg, patients with sPAP ≥ 40 mmHg more often had reduced LVEF (41.7% vs. 49.5%), elevated left ventricular filling pressure (43.9% vs. 21.6%), dilated left atrium (54.2% vs. 33.0%), mitral valve regurgitation (56.9% vs. 25.0%), tricuspid valve regurgitation (13.8% vs. 4.4%) and aortic valve stenosis (27.6% vs. 13.3%), Table 2.

### Outcome data

After 5-years of follow-up 86 patients died (all-cause mortality: 53.0%) while the 1-year all-cause mortality was 23.4% (38 deaths). Patients with sPAP ≥ 40 mmHg had a higher mortality rate, 5-year mortality rate of 69.6% (46 events) and 1-year mortality rate of 34.8% (23 events), compared to patients with sPAP < 40 mmHg, 41.6% (40 events) (*p* < 0.001) for 5-year mortality rate and 15.6% (15 events) (*p* = 0.004) for 1-year mortality rate.

### Association between sPAP and 1-year mortality

**Figure 1:**
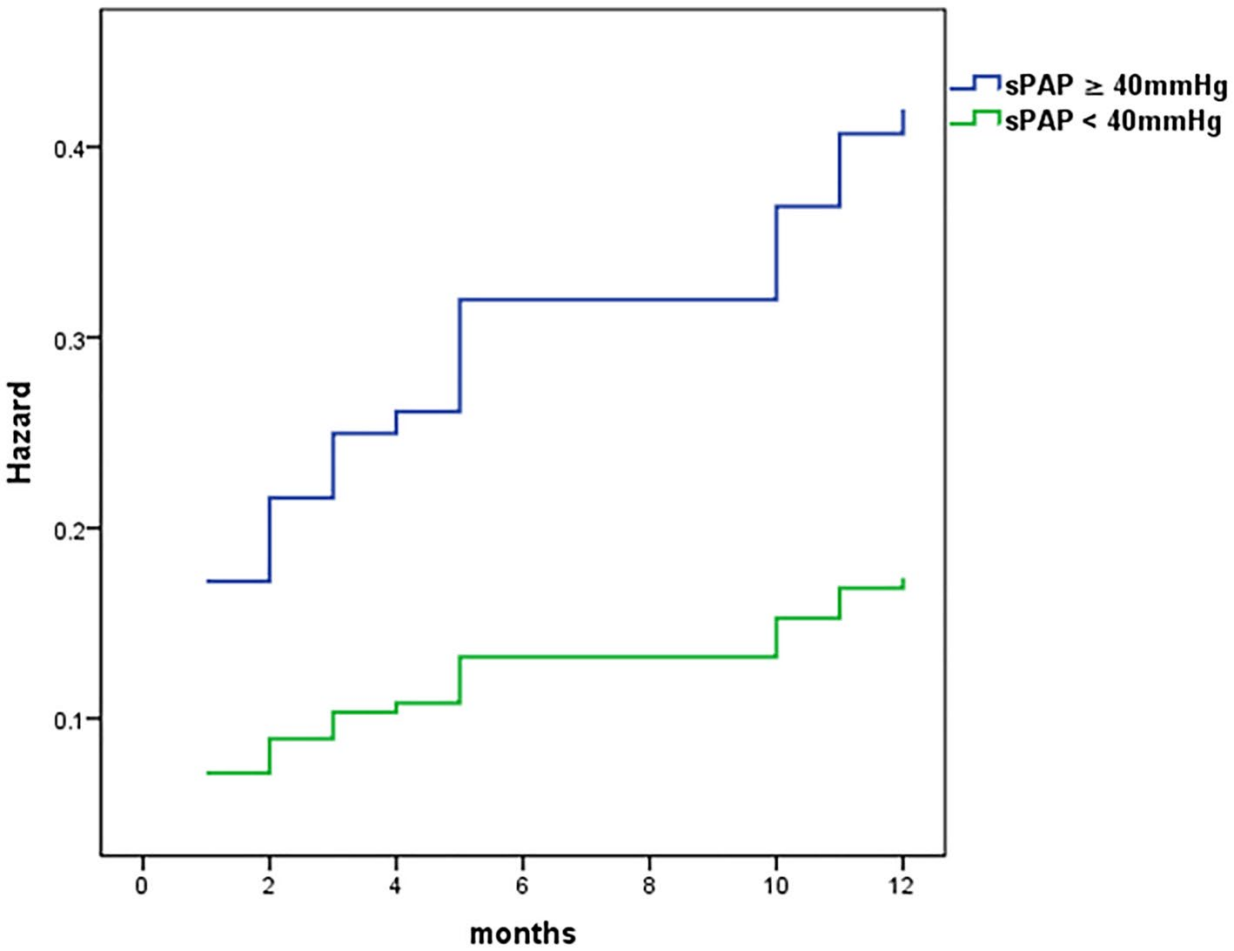
Adjusted 1-year risk of mortality, comparison between patients with sPAP ≥ 40 and patients with sPAP < 40 mmHg.

The association between sPAP and 1-year all-cause mortality is illustrated in Table 3. Multivariable analysis found a statically significant association between elevated sPAP and increased 1-year all-cause mortality rate with a cutoff level at ≥ 40 mmHg (HR 2.63, 95%CI 1.19–5.84, *p* = 0.017), Fig. 1. As a continuous variable, every increase of 5 mmHg in sPAP was associated with 17% increased relative risk for all-cause mortality (HR = 1.034, 95% of CI 1.004–1.065 and *p* = 0.025*)*.

**Table 3:** Univariate and multivariable Cox-regression analysis of factors for association with1-year all-cause mortality.

Variables	Univariable			Multivariable		
	HR	(95% CI)	*p*	HR	(95% CI)	*p*
Age, year	1.03	0.92–1.15	0.602	0.97	0.85–1.11	0.639
Gender, male	0.79	0.42–1.49	0.471	0.97	0.31–0.90	0.518
sPAP ≥ 40 mmHg	2.46	1.26–4.62	0.008	2.63	1.19–5.84	0.017
LVEF ≤ 45%	1.71	0.88–3.29	0.111	1.34	0.61–2.93	0.469
Diabetes Mellitus	1.26	0.57–2.75	0.570	1.17	0.49–2.77	0.727
Treatment with percutaneous coronary intervention (PCI)	0.56	0.29–1.09	0.086	0.72	0.33–1.6	0.424
Atrial fibrillation	0.89	0.39–2.05	0.791	0.78	0.31–1.96	0.601
Estimated glomerular filtration rate ≤ 35 ml/min	1.94	0.96–3.95	0.067	1.99	0.88–4.48	0.097

All variable in Tables 1 and 2 were included in the univariable models.

### Association between sPAP and 5-year mortality

**Figure 2:**
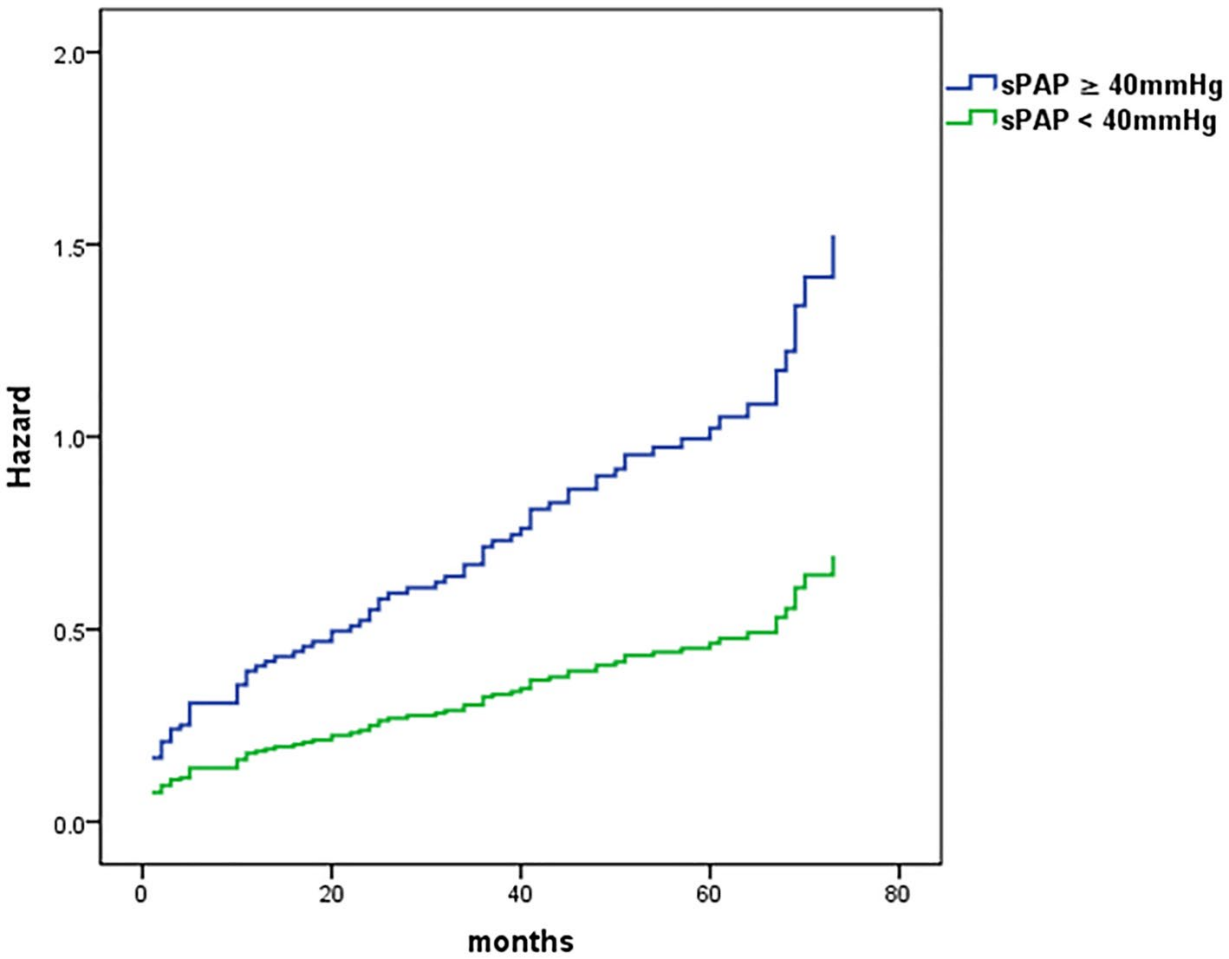
Adjusted 5-year risk of mortality, comparison between patients with sPAP ≥ 40 and patients with sPAP < 40 mmHg.

The association between sPAP and 5-year mortality is illustrated in Table 4. Cox proportional-hazard regression multivariable models adjusted for the baseline characteristics demonstrated a statistically significant association between elevated sPAP and increased all-cause mortality rate with a cutoff level at ≥ 40 mmHg (HR = 2.08, 95%CI 1.25–3.44, *p* = 0.005), Fig. 2. As a continuous variable, every increase of 5 mmHg in sPAP was associated with 15% increased relative risk for all-cause mortality (HR = 1.030, 95% of CI = 1.008–1.052 and *p* = 0.007*)*.

**Table 4:** Univariate and multivariable cox-regression analysis of factors for association with 5-year all-cause mortality.

Variables	Univariable			Multivariable		
	HR	(95% CI)	*p*	HR	(95% CI)	*p*
Age, year	1.09	1.01–1.17	0.021	1.02	0.93–1.11	0.693
Gender, male	0.97	0.64–1.49	0.891	1.9	1.1–3.2	0.019
sPAP ≥ 40 mmHg	2.21	1.44–3.38	< 0.001	2.08	1.25–3.44	0.005
LVEF ≤ 45%	1.26	0.82–1.92	0.293	1.42	0.85–2.438	0.158
Diabetes Mellitus	1.92	1.17–3.14	0.010	1.73	1.01–2.96	0.048
Treatment with percutaneous coronary intervention (PCI)	0.445	0.29–0.69	< 0.001	0.48	0.29–0.82	0.004
Atrial fibrillation	2.32	1.47–43.67	< 0.001	2.04	1.22–3.40	0.006
Estimated glomerular filtration rate ≤ 35 ml/min	2.19	1.36–3.54	0.001	2.35	1.37–4.01	0.006

All variable in Tables 1 and 2 were included in the univariable models.

*HR* Hazard ratio, *sPAP* Systolic pulmonary artery pressure, *LVEF* Left ventricular ejection fraction.

Also, male gender (HR 1.9, 95%CI 1.1–3.2, *p* = 0.019), atrial fibrillation (HR 2.04, 95%CI 1.22–3.40, *p* = 0.006) and eGFR ≤ 35 ml/min/1.73m^2^ (HR 2.35, 95%CI 1.37–4.01, *p* = 0.006) were associated with increased 5-year mortality rate. While treatment with percutaneous coronary intervention were associated with decreased 5-year mortality, (HR 0.48, 95%CI 0.29–0.82, *p* = 0.004).

## Discussions

The results of the present study demonstrate an association between elevated sPAP levels and one as well as five-year mortality rate after AMI in a cohort of elderly patients, all 80 years of age or older at the baseline. After multivariable adjustment, sPAP with a cutoff level at ≥ 40 mmHg was a strong independent predictor with a twofold increased risk for both one- and five-year all-cause mortality rate. Every increase of 5 mmHg in sPAP was associated with 17 and 15% increased relative risk for one- and five-year all-cause mortality, respectively.

To our knowledge, the present study is the first to analyze the impact of sPAP on one- and five-year prognosis after AMI in an elderly patient sample, ≥ 80 years. However, the impact of sPAP on survival after AMI have been demonstrated in several studied the last years^14–17^, but these studies compared with the present study were conducted in much younger patients with shorter follow-up periods. The results of the present study add more data to the available evidence supporting the impact of sPAP on both short- and long-term survival and in all patient age groups, including patients ≥ 80 years.

Interestingly, sPAP was a stronger predictor for both short- and long-term mortality, compared with LVEF which did not show any statistically significant impact in the Cox-regression multivariable models. These results indicate that sPAP as a marker of hemodynamic dysfunction^21^ after AMI is a stronger prognostic predictor, compared with the LVEF as a marker of structural LV dysfunction.

The multivariable Cox proportional-hazards regression models found sPAP ≥ 40 mmHg to be the only independent predictor for the one-year all-cause mortality after AMI. Whereas, independent predictors of increased five-year all-cause mortality, beside the sPAP ≥ 40 mmHg, were also diabetes mellitus and atrial fibrillation and eGFR ≤ 35 ml/min, while treatment with PCI had a survival protective effect. These results indicate that patients with elevated sPAP have a high risk for impaired survival already during the first year and these patients must be identified and tailored for secondary preventive managements as soon as possible.

### Pathophysiological mechanisms of elevated pulmonary artery pressure after AMI

AMI may results in decreased left ventricular (LV) pumping function and thereby increasing LV filling pressures. The increased LV filling pressures transmits backwards into the lung circulation, leading to an increase in the pulmonary artery pressure (PAP). The elevated PAP is frequently associated with a reactive increase in pulmonary vascular resistance (PVR), resulting in a further increase in PAP^22^. Thus, the pulmonary circulation after AMI is characterized by elevated PAP and PVR, which increases the afterload of the right ventricle (RV) and may contribute to RV dysfunction and eventually RV failure^23^.

The mechanism underlying the pulmonary vasoconstriction after AMI is not completely understood, but may involve alterations in angiotensin‐II^24–26^as well as endothelial dysfunction^22^.

The pulmonary vascular endothelium is the predominant site for the angiotensin-converting enzyme which hydrolyses angiotensin-I to angiotensin-II. The pulmonary circulation is very sensitive to the vasoconstrictive and proliferative effects of Angiotensin-II^24–26^, hence, after AMI develops progressive PHT and RVH with important pulmonary structural remodeling characterized by myofibroblasts proliferation and a vicious circle of cardiopulmonary dysfunction^26^.

One other reason for elevated PAP level could be ischemic mitral valve regurgitation, which is a common complication after AMI and often associated with poor prognosis^27–29^.

The above-mentioned evidence and mechanisms indicate that patients with elevated sPAP after AMI might benefit from tailored and intensive treatment with angiotensin enzyme inhibitors and angiotensin receptor blockers, in order to prevent the development of post AMI heart failure and thereby to improve survival.

### Conclusions

Elevated sPAP was an independent risk factor for one- and five-year all-cause mortality after AMI in very elderly patients and sPAP seems to be a better prognostic predictor for all-cause mortality than LVEF. The risk of all-cause mortality after AMI increased with increasing sPAP.

### Strengths and limitations

The data in the present study was collected from the medical records from any of the two largest cardiology centers in Gothenburg. All the echocardiography studies were performed of echocardiography specialist at the department of clinical physiology and all echocardiography reports were reviewed of the authors. However, in this observational study, medical records were studied retrospectively. In addition, despite our efforts in collecting as much information as possible, some patient data were not available. Besides, despite adjustment, we cannot rule out residual confounding from unmeasured variables. The sample size was relatively small and included patients with STEMI and patients with non-STEMI. There was a limited number of patients with adequate data on sPAP. Nevertheless, the study showed a significant association between elevated sPAP and mortality in patients of 80 years of age or older who had suffered a myocardial infarction.

Furthermore, sPAP estimation by echocardiography includes an approximation of the right atrial pressure using inferior vena cava width and its respiratory variation. The gold standard would be right heart catheterization for measurement of pulmonary artery pressure, a data which was not available in our present study.

### Clinical implication

In clinical practices after AMI, sPAP can be used as a marker of poor prognosis and a target in secondary preventive managements to reduce the mortality and morbidity rates. As secondary preventive managements after AMI, treatment with renin–angiotensin–aldosterone system (RAAS) inhibitors might improve the prognosis in patients with elevated sPAP after AMI. However, this is pure speculation. Treatment with ACEI/ARB had no significant impact on survival in the present study, which might be due to the fact that patients in the study had relatively low doses ACEI/ARB.

## Data Availability

The datasets used and/or analysed during the current study available from the corresponding author on reasonable request.

## Abbreviations

LVEF: Left ventricular ejection fraction
RA: Right atrium
sPAP: Systolic pulmonary artery pressure
AMI: Acute myocardial infarction
STEMI: ST-elevation myocardial infarction
Non-STEMI: Non-ST-elevation myocardial infarction
PCI: Percutaneous coronary intervention
IVC: Inferior vena cava
eGFR: Estimated glomerular filtration rate
BMI: Body mass index
BP: Blood pressure
ACEI: Angiotensin converting enzyme inhibitors
ARB: Angiotensin receptor blockers
HRs: Hazard ratios
CIs: Confidence intervals
