# Author Correction: Impact of elevated systolic arterial pulmonary pressure on the total mortality rate after acute myocardial infarction in the elderly

**DOI:** 10.1038/s41598-023-44930-w

**Published:** 2023-10-17

**Authors:** Salim Bary Barywani, Magnus C. Johansson, Silvana Kontogeorgos, Zacharias Mandalenakis, Per-Olof Hansson

**Affiliations:** 1 Department of Molecular and Clinical Medicine, Institute of Medicine, Sahlgrenska Academy, University of Gothenburg, Gothenburg, Sweden; 2 Department of Medicine, Geriatrics and Emergency Medicine, Region Västra Götaland, Sahlgrenska University Hospital, Gothenburg, Sweden; 3 Department of Clinical Physiology, Region Västra Götaland, Sahlgrenska University Hospital, Gothenburg, Sweden

Correction to: *Scientific Reports* 10.1038/s41598-022-16210-6, published online 23 July 2022

The original version of this Article contained an error in the spelling of the author Silvana Kontogeorgos which was incorrectly given as Silvana Kontogergos.

The original Article has been corrected.

